# Serological Weak Expression of D Antigen: A Retrospective Study of Blood Donors and Patients at a Teaching Hospital in Eastern India

**DOI:** 10.7759/cureus.75125

**Published:** 2024-12-04

**Authors:** Suman S Routray, Gopal K Ray, Nirupama Sahoo, Bakki Israel Karunakar, Sukanta Tripathy, Devi Acharya

**Affiliations:** 1 Immunohematology and Blood Transfusion, Kalinga Institute of Medical Sciences, Bhubaneswar, IND; 2 Transfusion Medicine, All India Institute of Medical Sciences, Guwahati, IND

**Keywords:** anti-d, anti d reagent, del phenotype, d typing, partial d, rh d antigen, rh-hr blood-group system, rh immunization, weak d

## Abstract

Background and objective

RhD variants show altered D antigen expression, affecting their serological detection. Proper identification is crucial due to potential anti-D antibody formation. This study aimed to retrospectively analyze the frequency and characteristics of D variant cases encountered during RhD typing in both blood donors and recipients and the transfusion implications.

Methods

We conducted a retrospective analysis of the D variant involving all the donors and patients whose samples were tested for blood grouping. RhD typing was done using monoclonal anti-D reagents via conventional tube technique (CTT) and column agglutination technique (CAT). Weak reactions (≤ 2+) were retested with different antisera. Weak D (Du) testing was conducted on serologically negative RhD results in donors. D variants were suspected based on discrepancies between CTT and CAT, weak reactions with different antisera, positive Du testing in RhD-negative donors, or anti-D alloimmunization in RhD-positive individuals. Data are presented in numbers and percentages. The odds ratio (OR) determining the association between different blood groups and age groups with the D variant in the donor population was calculated. A p-value <0.05 was considered statistically significant.

Results

D variants were found in 0.11% of donors and 0.039% of patients, with 21.7% being females. In the patient population, all the D variant cases were from surgical patients with transfusion requests; three received RhD-negative units, while seven did not require transfusions. D variants were more common in adult donors (25-44 years), with an 8.5 times higher occurrence in the AB group compared to the A group.

Conclusions

The D variant has a high prevalence in eastern India. Regional centers should be equipped to accurately identify and differentiate D variants, enabling improved management and effective conservation of RhD-negative units.

## Introduction

The Rh blood group system consists of a complex array of more than 56 antigens and holds significant clinical relevance in transfusion medicine [1]. Among these, the D antigen is the most common and immunogenic, encoded by the polymorphic RhD gene. This gene contains over 460 alleles, and mutations can alter the expression of the D antigen, categorized as D variants. These variants are typically classified into three categories - weak D, partial D, and DEL - based on their genotype and their capacity to produce allo-anti-D antibodies.

The weak D variant expresses a reduced but complete form of the D antigen. This diminished expression is typically caused by a missense mutation affecting the transmembrane or intracellular regions of the RhD protein, resulting in 150 distinct weak D types [2]. Of note, weak D types 1, 2, and 3 are unlikely to generate anti-D antibodies because the conformational changes in their RhD protein occur inside the red blood cell. Furthermore, steric hindrance resulting from the spatial arrangement of the C antigen relative to the D antigen, when a Rh Ce (r') gene is trans to a normal RhD gene, can also lead to decreased expression of the D antigen on the RBC surface [3].

In contrast, partial D represents a qualitative disorder resulting from missense mutations or genetic conversions that result in the loss of one or more epitopes on the D protein. This leads to altered reactivity with different anti-D typing reagents. Recently, the International Society of Blood Transfusion (ISBT) has acknowledged the presence of a "weak partial" variant, which reflects qualitative changes in the epitopes of weak D types and is prone to D alloimmunization. This category includes weak D types 4.2, 11, 15, and 21 [4]. The DEL phenotype is a weak variant of D that can only be detected through the adsorption and elution of anti-D from red cells that do not show a positive serological reaction with anti-D. This phenotype is particularly prevalent in East Asian populations, with approximately 98% of DEL phenotypes attributed to the RHD*DEL1 or RHD*01EL.01 alleles [5]. Due to the extremely low number of antigenic sites, conventional reagents often fail to detect DEL, making adsorption and elution the primary methods for its identification.

Characterizing D variants is clinically essential, as the transfusion of D-positive units to individuals with these variants can provoke anti-D antibody production or induce alloimmunization in D-negative recipients [6]. In India, where molecular typing facilities are limited, serological suspicion remains the cornerstone of managing D variants. Geographic and ethnic diversity further influences the estimated prevalence of weak D among blood donors in India, with rates ranging from 0.0075% to 0.2% [7]. The DEL phenotype has a prevalence of approximately 0.2% among D-negative North Indian donors, though it may reach up to 2.8% in D-C+ individuals [8]. This study aims to analyze the frequency and characteristics of D variant cases identified during RhD typing in both blood donors and recipients in 2023. By examining cases with weak or discrepant RhD typing results, the study seeks to identify trends in testing and improve the management of RhD variant cases, ultimately enhancing strategies to prevent alloimmunization and transfusion reactions.

## Materials and methods

Study design and population

A retrospective analysis was conducted using paper records from a tertiary care hospital in Odisha, covering the period from January 1, 2023, to December 31, 2023. A total of 36,350 blood samples, from both blood donors and patients, were examined during the study period. The study included two populations: blood donors and patients requiring blood typing for transfusion support. All donors and patients whose samples were tested for blood group were included in the study. These individuals underwent standard RhD typing, with additional tests conducted when discrepancies were observed. Samples from individuals with known blood disorders were excluded. The study was approved by the Institutional Ethics Committee (IEC) of KIIT University (approval no: KIIT/KIMS/IEC/1899/2024, dated September 18, 2024).

RhD typing procedures

Initial RhD typing was performed using two methods: the column agglutination technique (CAT) and the conventional tube technique (CTT). CAT uses blood grouping gel cards (Tulip Diagnostics Pvt. Ltd., St. Cruz, India) to detect the RhD antigen on red blood cells by observing agglutination in the gel column after exposure to pre-filled anti-D antisera. CTT uses monoclonal anti-D antisera to identify the RhD antigen. The strength of agglutination was graded according to standard AABB methods as summarized in Table 1. In cases with discrepancies in reaction strength between CTT and CAT, or where the initial RhD reaction strength was ≤2+ in either method, RhD typing was repeated using CTT with monoclonal anti-D antisera from two different lots, if necessary. This supplementary confirmatory test was used to resolve discrepancies in the initial typing.

**Table 1:** Grading of strength of agglutination by conventional tube technique

Strength of agglutination	Definition in tube method	Definition in gel card
4+	One solid agglutinate	Agglutinates are at the top of the gel column
3+	Several large agglutinates	Agglutination is spreading from the top of the gel column to half of the column
2+	Medium-size agglutinates, with a clear background	Agglutination is spreading throughout the column from the bottom
1+	Small-size agglutinates with turbid background	Agglutinates just spread from the bottom to the maximum half of the column

Specific Testing for Blood Donors

Blood donors who tested RhD-negative by both CAT and CTT underwent additional weak D (Du) testing. The Du test was performed during the indirect antiglobulin test (IAT) phase, using blended IgM and IgG anti-D antisera at 37 °C, following the recommended protocol. This test detects weak D antigens in individuals who are serologically RhD-negative but carry partial or variant RhD antigens.

Specific Testing for Patients

For patients with inconclusive results, Du testing was performed to detect the presence of the D antigen. Du testing was not performed on patients who tested RhD-negative by both CAT and CTT, as these patients were assigned RhD-negative, ABO-compatible blood units.

Identification of D variant cases

A D variant was suspected when we observed any of the following: a) weak reaction strength (≤2+) with anti-D antisera from two different lots in both CAT and CTT; b) discrepancies in reaction strengths between CTT and CAT methods; c) RhD-negative results by both CAT and CTT but positive Du testing in blood donors; and d) detection of anti-D alloimmunization in individuals who were serologically RhD-positive.

Data analysis

The data were analyzed using SPSS Statistics version 19 (IBM Corp., Armonk, NY). Descriptive statistics were used to summarize the characteristics of weak D or variant RhD cases, and frequency analysis was conducted to determine the incidence of D variant cases among blood donors and recipients. The prevalence odds ratio (OR) by Fisher's exact test was employed to calculate the strength of association between blood groups and D variants; a p-value <0.05 was considered statistically significant.

## Results

A total of 23 cases of D variants were detected serologically out of 36,350 blood typing performed. The distribution of gender, age, and blood group among donor and patient cases is summarized in Table 2.

**Table 2:** Distribution of D variant cases in donor and patient populations

	Donor population		Patient population		Total cases of D variant
	N	D variant, n (%)	N	D variant, n (%)	
Total	11,005	13 (0.11)	25,345	10 (0.04)	23
Gender					
Male	10,835	12 (0.1)	13,341	6 (0.05)	18
Female	170	1 (0.01)	12,004	4 (0.03)	5
Age (years)					
<17 (pediatrics)	0	0	3,558	2 (0.05)	2
18-24 (young adults)	2,861	4 (0.03)	2,299	2 (0.09)	6
25-44 (adults)	7,483	7 (0.06)	8,772	3 (0.03)	10
45-59 (middle-aged adults)	661	2 (0.02)	6,059	1 (0.02)	3
>60 (older adults)	0	0	4,657	2 (0.04)	2
Blood group					
A	2,414	1 (0.01)	5,328	3 (0.06)	4
B	3,578	2 (0.02)	8,370	3 (0.03)	5
AB	843	3 (0.03)	1,772	1 (.06)	4
O	4,167	7 (0.06)	9,875	10 (0.1)	17

Patient population

Weakly expressed D status (weak+ to 2+ by CAT) was found in 10 of the 25,345 patients tested. In CTT, using different lots of anti-D reagents, reactions varied from 1+ to no reaction but showed a strength of 2+ to 3+ reaction in the IAT phase. The distribution of cases was even across blood types, with A, B, and O each having three cases, and AB having one. All suspected cases belonged to surgical fields with transfusion needs, and only three required transfusions with group-specific RhD-negative units. Notably, D variants were identified in three females of reproductive age (15-49 years).

Donor population

Nine of the 10,630 RhD-positive units screened by CAT had weakened strength (less than 2+), while a 3+ strength was seen in the IAT phase with a monoclonal blend of anti-D reagent. Additionally, among the initial 375 RhD-negative units tested (no reaction in CAT and CTT), four cases showed 1+ to 2+ strength reactions in the IAT phase of Du testing. In total, 13 D variants were observed among the 11,005 donor units screened, yielding a prevalence of approximately one in 1,000 healthy donors. The D variants were predominantly found in adults aged 25-44 years (n = 7), although no significant association was noted between age groups and D variants. A significant association between the blood group and the D variant was observed, with the odds of a D variant being 8.5 times higher in the AB group compared to the A group, as depicted in Table 3.

**Table 3:** Association of blood groups and age groups with D variants among blood donors

Blood group	Total number of donors	Cases of D variant	Prevalence odds ratio
A	2,414	1	1
B	3,578	2	1.349
O	4,167	7	4.055
AB	843	3	8.591
Bombay	3	0	-
Age group (years)			
18-24	2,861	4	1
25-44	7,483	7	0.669
45-60	661	2	2.168

## Discussion

The prevalence of D variants in Indian studies has primarily focused on blood donor populations. Our study highlights the variation in distribution, revealing rates of 0.11% in donors and 0.039% among patients. This contrasts with previous studies from Eastern India, which reported only three cases of weak D and a 0.097% prevalence in South Indian donors [9, 10]. Variations in definitions, reagent types, and RhD typing methods significantly influence the interpretation of D variants. The reagents used for D typing can differ widely, and commercially available anti-D reagents fail to detect all cases of D variants. One study proposed using two anti-D reagents from different cell lines, specifically LHM 70/45 and one from the combination of LHM-76/59, LHM-76/55, and ESD-1, to aid in better detection of D variants in the Indian population during RhD typing [11, 12]. British guidelines recommend specific reagents for patient D typing that do not react with the DVI variant, which is commonly associated with anti-D production [13]. The need for developing indigenous testing strategies tailored to the Indian population is emphasized by findings of unique weak D types prevalent in local demographics [14].

Notably, females accounted for 21.7% of all D variants in our study, underscoring the importance of identifying these variants in women of reproductive age. Mistyping can increase the risk of sensitization, adversely affecting obstetric outcomes and subsequent pregnancies [15]. Rh immunoglobulin prophylaxis varies depending on the D variant type [16], and a Canadian study found significant rates of mistyping among pregnant women [17]. D variants were more frequent among donors aged 25-44 years, correlating with the typical age range of blood donors at our center. Conversely, D variants were detected more frequently in older patient populations. Disease states and aging may influence Rh protein expression, resulting in weakened D antigen expression. Interestingly, D variants were predominantly identified among O blood group donors at our center, contrasting with a higher prevalence reported in the B blood group in South India [18].

The cases of weakened D expression identified in the patient population were all linked to surgical cases requiring transfusion support. In the absence of molecular typing, addressing these needs becomes challenging. We provided D-negative units to three young adult patients due to sufficient inventory availability. However, the policy of administering RhD-negative units to individuals with D variants without molecular characterization strains the inventory of rare units and complicates management when adequately tested negative units are unavailable. Older males and females may receive ABO-compatible D-positive units during times of insufficient inventory. However, administering RhD-positive units to individuals with D variants poses a risk of alloimmunization, necessitating further surveillance.

The retrospective design of this study presents a limitation, as it may have contributed to the underreporting of D variants. Additionally, there is a lack of data regarding the prevalence of D variants across different ABO blood groups and age groups. Notably, our study identified a significant occurrence of D variants among AB group donors, which warrants further investigation. Future research should aim for larger sample sizes and adopt prospective designs to provide a more comprehensive understanding of D variant prevalence and its clinical implications.

## Conclusions

This study reports a high prevalence of weak D phenotype expression in blood donors (0.11%) and patients (0.039%) from eastern India, with a notable frequency of D variants in the donor population and surgical patients. Our findings emphasize the need for the development of accurate detection methods for D variants, which should be integrated into clinical practice to inform national transfusion guidelines, thereby preventing alloimmunization and its adverse consequences.
